# Cross-sectional computed tomography imaging of splenic hamartoma

**DOI:** 10.11604/pamj.2025.52.151.44907

**Published:** 2025-12-09

**Authors:** Devyansh Nimodia, Pratapsingh Hanuman Parihar

**Affiliations:** 1 Department of Radiodiagnosis, Datta Meghe Institute of Medical Sciences, Sawangi, Wardha, Maharashtra, India

**Keywords:** Hamartoma, rare, hyperdense

## Image in medicine

A 21-year-old male residing in the Southeast Asia region presented to the emergency department with intermittent left upper quadrant pain and mild fatigue lasting for several weeks with vague complaints of fever with 3-4 episodes of loose stool and multiple episodes of vomiting. He had no significant past medical history and was not on any medications. Physical examination revealed mild tenderness in the left upper quadrant but no palpable mass. He was advised a contrast-enhanced computed tomography (CECT) examination which revealed hyperdense (A) enhancing lesion noted at lower pole of spleen which is enhancing in arterial (B) phase with rapid washout on portal (C) and delayed phase (D). The lesion is noted to get arterial feed from splenic artery, suggestive of splenic hamartomas. Splenic hamartomas are very rare lesions commonly found incidentally on imaging. They are most often solitary but may be present as multiple nodules in patients with tuberous sclerosis or Wiskott-Aldrich syndrome. Splenic hamartomas are very rare, with only 3 described in a series of 200,000 splenectomies. Hamartomas are normally incidental findings at imaging, surgery, or autopsy. They can occur in any age group. Symptoms occur from mass effect if they grow large. Splenic hamartomas may not require surgical intervention unless they are large or symptomatic. Imaging characteristics can be misleading, and histopathological examination remains the gold standard for diagnosis. In this case, the patient's symptoms were attributed to the hamartoma, which was successfully managed with splenectomy.

**Figure 1:**
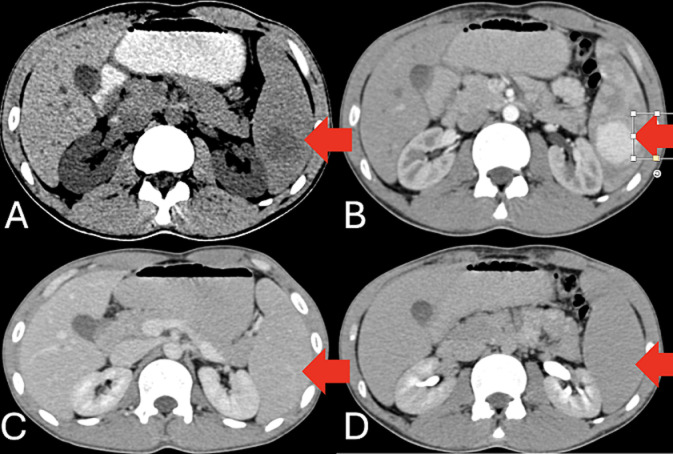
contrast-enhanced computed tomography image of abdomen in axial section showing hyperdense (A); enhancing lesion noted at lower pole of spleen which is enhancing in arterial (B); phase with rapid washout on portal (C) and delayed phase (D) suggestive of splenic hamartoma

